# Does smoking status affect the likelihood of consulting a doctor about respiratory symptoms? A pilot survey in Western Australia

**DOI:** 10.1186/1471-2296-10-16

**Published:** 2009-02-17

**Authors:** Moyez Jiwa, Hayley Arnet, Georgia Halkett, Marthe Smith, Moira O'Connor, Julia Rhodes, Kate Poland, Max Bulsara

**Affiliations:** 1 Western Australian Centre for Cancer and Palliative Care, Curtin University of Technology, Perth, Western Australia, Australia; 2 University of Western Australia, Perth, Western Australia, Australia

## Abstract

**Background:**

Smokers attribute respiratory symptoms, even when severe, to everyday causes and not as indicative of ill-health warranting medical attention. The aim of this pilot study was to conduct a structured vignette survey of people attending general practice to determine when they would advise a person with respiratory symptoms to consult a medical practitioner. Particular reference was made to smoking status and lung cancer.

**Methods:**

Participants were recruited from two general practices in Western Australia. Respondents were invited to complete self-administered questionnaires containing nine vignettes chosen at random from a pool of sixty four vignettes, based on six clinical variables. Twenty eight vignettes described cases with at least 5% risk of cancer. For analysis these were dubbed 'cancer vignettes'. Respondents were asked if they would advise a significant other to consult a doctor with their respiratory symptoms. Logistic regression and non-parametric tests were used to analyse the data.

**Results:**

Three hundred questionnaires were distributed and one hundred and forty completed responses were collected over six weeks. The majority (70.3%) of respondents were female aged forty and older. A history of six weeks' of symptoms, weight loss, cough and breathlessness independently increased the odds of recommending a consultation with a medical practitioner by a factor of 11.8, 2.11, 1.40 and 4.77 respectively. A history of smoking independently increased the odds of the person being thought 'likely' or 'very likely' to have cancer by a factor of 2.46. However only 32% of cancer vignettes with a history of cigarette smoking were recognised as presentations of possible cancer.

**Conclusion:**

Even though a history of cigarette smoking was more likely to lead to the suggestion that a symptomatic person may have cancer we did not confirm that smokers would be more likely to be advised to consult a doctor, even when presenting with common symptoms of lung cancer.

## Background

Respiratory symptoms, such as coughing and breathlessness, are common reasons for people to seek advice from their General Practitioner (GP). For example, GPs in Australia will see more than six patients with a cough for every 100 patient consultations [1]. However, less than two percent of patients who present to their GP with an ongoing cough will be diagnosed with cancer [2]. To press most cases of lung cancer are diagnosed among symptomatic patients rather than coincidentally or through screening tests. Survival from lung cancer depends on access to a specialist when the disease is treatable. However, as is noted in the literature, some patients, with a history of cancer symptoms delay visiting their GP. This may be due, in part, to a lack of recognition of risk factors for serious illness including cancer [3]. Factors that promote help seeking via medical consultation are: associating symptoms with cancer and being encouraged to consult by significant others [4].

Cigarette smoking is the most significant risk factor for lung cancer. Smokers frequently develop respiratory symptoms. Corner at al. reported that smokers attributed their symptoms, even when severe, to everyday causes and not as indicative of ill-health warranting medical attention. In some cases smokers were unsure whether the symptoms they were experiencing were 'normal' and related to smoking, rather than something that should be presented to a doctor [5]. In a related study lung cancer patients recalled having new symptoms for many months before their diagnosis, irrespective of their disease stage once diagnosed. Chest symptoms (cough, breathing changes, and pain in the chest) were common, as were systemic symptoms (fatigue/lethargy, weight loss and eating changes). Although symptoms were reported as being marked changes in health, only haemoptysis was interpreted as serious enough to warrant urgent medical attention by patients [6]. Patients in the UK were invited to rate a series of symptoms to the extent that they were 'cancer' symptoms (knowledge) and interviewed about whether the symptoms would prompt them to visit a doctor (hypothetical help seeking behaviour). The majority of patients had 'fair' knowledge about cancer symptoms [7]. More information is needed to understand the factors that influence the decision to consult medical practitioners.

Clinical Judgement analysis offers a quantitative method of probing the judgments of patients and to identify systematic differences in their perceptions of risk and benefit [8]. The technique includes the presentation of vignettes and has a major advantage of allowing comparison of different respondents' behaviours over the same set of cases and estimating the independent effects of specific information on a person's judgements[9]. The aim of this pilot study was to conduct a structured vignette survey of people attending general practice to determine when they would advise a person with respiratory symptoms to consult a medical practitioner. Particular reference was made to smoking status and lung cancer.

## Methods

### Ethics

This study received ethics approval from HREC at the University of Western Australia (RA/4/1/1459). Return of the completed questionnaire was considered consent to participate in the survey.

### Setting and recruitment

The practices were located in north metropolitan Perth where up to 95 practices are organised under a single 'division' of general practice. Two general practices were recruited from six approached to distribute the questionnaires to patients in their waiting rooms over a six-week period. Each practices had a catchment area of 14,000 patients and saw approximately 500 patients per week. Questionnaires were left at the reception desk with a general invitation to participate in the survey. Respondents were people waiting to consult a GP for a routine appointment at the practice. People aged over 18 years were eligible to participate.

### Vignettes and randomisation

Study participants were presented with self-administered questionnaires incorporating 'vignettes' (short stories) about people who were experiencing respiratory symptoms. The questionnaire was piloted with twenty respondents to ascertain ease of completion. Minor changes to the text and formatting were adopted following this review. Each vignette was constructed with six clinical details with two possible variations. The sample size required is a factor of the number of variables modeled as discussed under sample size calculations. With six variables there were 64 (2^6^) potential scenarios to cover each of the possible combinations. [9] The vignettes focused on symptoms of respiratory disease as potential reasons to consult a doctor. The vignettes were presented to the sample in an 'incomplete-within-blocks' design, in other words while each individual is not exposed to every possible combination of the vignette characteristics, within blocks of respondents this can be achieved. It was thus possible to reduce the number of vignettes presented to each respondent to nine [10].

**Figure 1:**
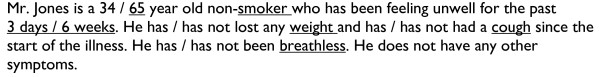
**Example of vignette**. Indirect variables underlined.

Vignettes for 'cancer' patients were based on symptom profiles considered to have a greater than 5% risk of lung cancer [11]. The choice of symptoms e.g. cough, breathlessness and weight loss was based on the relative frequency of such symptoms in the community [12]. The cases that were identified by the physician members of the team as warranting an urgent medical consultation were cases with a greater than 5% risk of cancer based on the particular combination of symptoms in each vignette and with reference to published guidelines [11]. They were dubbed 'cancer' vignettes in this study. Twenty-eight of the sixty-four vignettes described such cases. Each scenario was presented as a story, as depicted in Figure 1.

### Questionnaire

**Figure 2:**
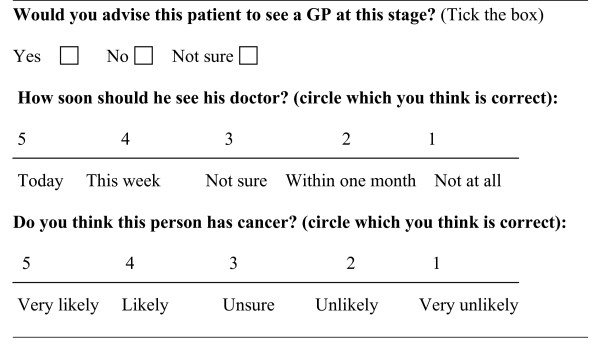
**Questions to participants**.

Respondents were asked what advice they would give the person in each of the vignettes. See Figure 2. The following demographic data was collected: age, gender, country of birth, ethnicity and smoking status.

### Sample size and statistical analysis

130 respondents were required for binary logistic regression with 10 explanatory variables. The formula applied was: sample size = 50 + 8 × number of variables for 80% power [13]. Logistic regression was used to examine three outcomes; the odds of recommending a consultation, the odds of recommending a consultation within a week and the odds of stating that a scenario was a cancer presentation. We modelled the age of the patient in the vignette, smoking status, duration of symptoms, cough, weight loss and breathlessness. Also included in the model were respondent characteristics including age, gender, smoking status and ethnicity. In order to control for clustering by individual respondent a cluster option was used to estimate robust standard errors. Non-parametric tests were applied to further explore the relationship between the clinical symptoms described in the vignette and the advice to seek an appointment 'today'.

## Results

One hundred and fifty individual questionnaires were made available at each of the two practices. 140 completed questionnaires were collected after six weeks. Questionnaires were left in a prominent location at the reception desk, it was not possible to ascertain how many people considered participating, but then returned the survey uncompleted. All participants responded to each of the nine vignettes presented. Each vignette was presented on average 19.68 times (Range 9–32). All the vignettes were incorporated into the analysis. The majority of respondents were female (70%) and the vast majority (80%) considered themselves Australian or European. The demographics of the sample, majority older females, were consistent with those of consulting patients reported previously [14]. See Table 1, 'Demographic characteristics' in additional files.

In most vignettes, where respondents expressed a view (1202/1260), they indicated that the patient should consult a GP (1056/1202) 83.8%. The majority who answered the question (952/1228) or 77.5% recommended a consultation within a week and (215/1228) or 17.4% suggested an appointment today.

### Recommending a consultation with a General Practitioner in the case as described

None of the respondent variables appeared to influence which cases would be advised to consult a GP. Of the clinical details incorporated in the vignettes, six weeks of symptoms, breathlessness or weight loss were more likely to lead to this suggestion. Table 2 displays the extent to which the variables influenced the outcome variable. Twenty-one percent of the variability could be explained from these independent variables. Area under ROC curve = 0.83. Therefore the accuracy of model as a test for respondents' views could be described as 'good'. The sensitivity of the model was 99.5%, specificity 17.8%, positive predictive value was 91.2% and the negative predictive value was 68.4%.

### Recommending an appointment with a doctor within one week

For the purposes of this study we assumed that seeking an appointment within one week was a mark of significant concern for the patient described in the vignette. Respondent characteristics did not appear to have a bearing on the decision. The most significant features were six weeks of symptoms, breathlessness and or weight loss. See Table 2. Twenty one percent of the variability could be explained from these independent variables. Area under ROC curve = 0.80. Therefore the accuracy of model as a test for respondents' views could also be described as 'good'. The sensitivity of the model was 96.2%, specificity 34.5%, positive predictive value was 84.9% and the negative predictive value was 67.4%.

### Identifying potential cancers

Respondents performed less well in identifying potential 'cancers' explicitly. Cancer vignettes were presented five hundred and eight times in the survey. Respondents suggested the patient is 'likely' or 'very likely' to have cancer and should make an appointment within one week in one hundred and twenty two occasions (24%). In more than half of all cases (53%) respondents were unsure of the chance that the symptoms were related to cancer. Of the cancer vignettes 55% included a history of cigarette smoking. Only 32% of cancer vignettes with a history of smoking were recognised as 'likely' or 'very likely' to have cancer. Cancer was suspected in 13.8% of all vignettes However, most cases respondents identified as cancer (77.5%) would be classified as 'high risk' on current medical guidelines. Twenty six per cent of the variability could be explained from these independent variables. Area under ROC curve was 0.85. Therefore the accuracy of model as a test for respondents' views could be described as 'good'. The sensitivity of the model was 32.5%, specificity 95.3%, positive predictive value was 52.4% and the negative predictive value was 89.8%. The most significant symptoms were duration of symptoms, weight loss, breathlessness, symptomatic person's smoking status, age and cough in that order. See Table 2, 'The impact of variables on respondents' decisions' in additional files.

## Discussion

Overall, in relation to the vignettes, most respondents recommended an appointment with a doctor and almost one in four recommended an appointment 'today'. While most cases that respondents believed described symptoms of cancer did indeed describe patients at high risk, we found no evidence that respondents could recognise most of these cases as potentially cancer presentations, even with a history of cigarette smoking. The odds of recommending an attendance at a GP within one week was more related to symptoms than smoking status. This was surprising as one would have anticipated that respondents who were also smokers were more aware of the need for vigilance about symptoms. The association between smoking and cancer has long been established and is widely known to the public. [15]

Our findings emphasise the need for strategies to influence significant others in advising symptomatic patients who require urgent medical advice. Smith, Pope and Botha reported a search of international publications (1985–2004) for delay in cancer diagnosis and identified common themes across the studies [16]. They remark on the important role of friends, family, and health-care professionals in the sanctioning of consultation. Clearly some people in this survey might issue significant others with inappropriate advice. However we urge caution when applying our data to an individual case. We were only able to incorporate a limited list of symptoms and did not include other potentially significant factors including local access to medical practitioners. The modest regression coefficients confirm that there may be other clinical or respondent characteristics that influence the decision to recommend consultation. We also acknowledge the unquantifiable biases inherent in distributing surveys at a reception desk. In future studies we recommend enlisting practice staff in distributing the questionnaires so that one can more comprehensively record the number of people who might have been eligible for the study.

Secondly, the representativeness of the vignettes was not formally established. We recognise that the symptoms of lung cancer can be vague and that some patients have no chest symptoms, and instead present with lethargy, weakness or weight loss [6]. The impact of many of these symptoms was not modelled in this study. Hamilton et al. found eight signs and symptoms that indicated the development of lung cancer and several of these were not apparently related to the respiratory tract [17]. Any follow up study would also need to include a robust theoretical framework. The Theory of Planned Behaviour posits that individual behavior is driven by behavioral intentions where behavioural intentions are a function of an individual's attitude toward the behaviour, the subjective norms surrounding the performance of the behavior, and the individual's perception of the ease with which the behavior can be performed (behavioral control). Attitudes toward the behavior is defined as the individual's positive or negative feelings about performing a behaviour [18]. Many of these parameters were not addressed in this pilot study but may be usefully incorporated into future studies.

## Conclusion

When recommending a consultation with a general practice our respondents were hesitant or unable to suggest a diagnosis of cancer. While it was encouraging that a history of cigarette smoking is more likely to lead to a suggestion of cancer in the context of significant symptoms many cases are not recognized even when smoking is a feature. It is important that those who might advise friends and relatives about symptoms are aware of the increased risk of cancer in the context of cigarette smoking and discourage procrastination.

## Competing interests

The authors declare that they have no competing interests.

## Authors' contributions

MJ designed the study and co-authored the paper, HA, GH and MS designed the study and co-authored the paper, MO'C co-authored the paper, KP and JR, arranged data collection and data entry. MB analysed the data. All authors read and approved the final manuscript.

## Pre-publication history

The pre-publication history for this paper can be accessed here:


